# Supplementary material for: Reversible control over the distribution of chemical inhomogeneities in multiferroic BiFeO3
Source: Nat Commun. 2025 Apr 27;16:3951. doi: 10.1038/s41467-025-59044-2 (PMC12034765; doi:10.1038/s41467-025-59044-2)
Supplement: Supplementary file 1 — Supplementary Information [file 41467_2025_59044_MOESM1_ESM.pdf]

Supplementary Information

Reversible control over the distribution of chemical inhomogeneities in multiferroic $BiFeO_3$ system

Authors: M. Müller[1], B. Yan[1], H. Ko[1], Y.-L. Huang[2–4], H. Lu[5,6], A. Gruverman[5,6], R. Ramesh[3,4,7–9], M. D. Rossell[10], M. Fiebig[1], and M. Trassin[1]

Affiliations:

[1] Department of Materials, ETH Zurich; Zurich, 8093, Switzerland.

[2] Department of Materials Science and Engineering, National Yang Ming Chiao Tung University; Hsinchu, 30010, Taiwan.

[3] Department of Materials Science and Engineering, University of California, Berkeley; Berkeley, 94720, USA.

[4] Materials Science Division, Lawrence Berkeley Laboratory, Berkeley; Berkeley, 94720, USA.

[5] Department of Physics and Astronomy, University of Nebraska; Lincoln, NE 68588, USA

[6] Nebraska Center for Materials and Nanoscience, University of Nebraska; Lincoln, NE 68588, USA

[7] Department of Physics, University of California, Berkeley; Berkeley, 94720, USA.

[8]Department of Materials Science and Nanoengineering, Rice University; Houston, 77005, USA.

[9]Department of Physics and Astronomy, Rice University; Houston, 77005, USA.

[10] Electron Microscopy Center, Empa, Swiss Federal Laboratories for Materials Science and Technology; Dübendorf, 8600, Switzerland.

1. X-ray diffraction on a $La_{0.15}Bi_{0.85}FeO_3/SrRuO_3/DyScO_3$ heterostructure

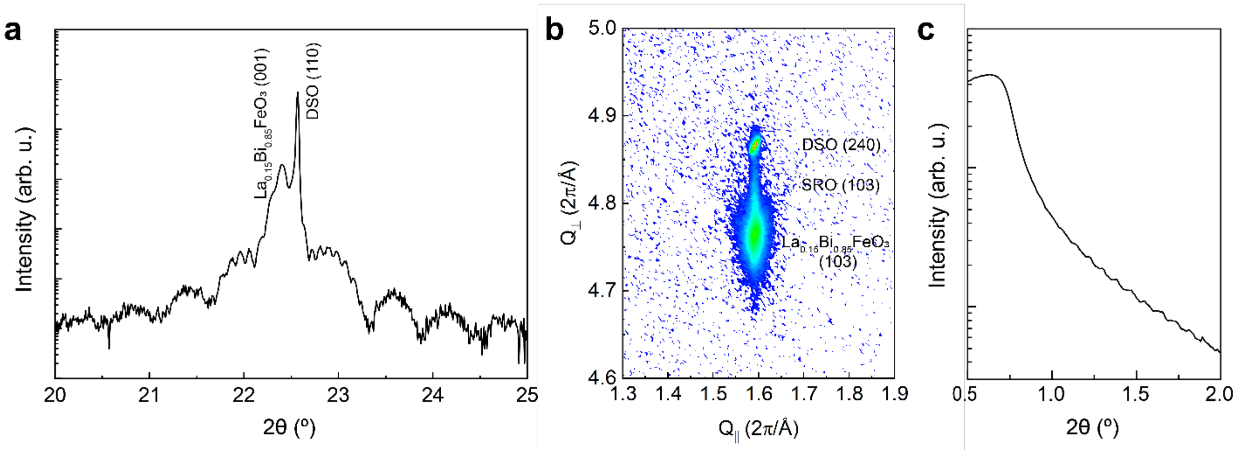

Fig. S1: X-ray diffraction on a $La_{0.15}Bi_{0.85}FeO_3/SrRuO_3/DyScO_3$ heterostructure. a, Symmetric θ−2θ diffractogram of a $(001)_{p.c.}$-oriented $La_{0.15}Bi_{0.85}FeO_3$ film. The presence of Laue fringes shows the high quality of the thin film, and their periodicity reveals the thickness of the $La_{0.15}Bi_{0.85}FeO_3$ film as 100 nm, whereas the $SrRuO_3$ buffer layer is 14 nm thick. **b,** Reciprocal space map (out-of-plane $Q_\perp$ and in-plane $Q_\parallel$) around the DSO $(240)_o$ reflection. The $La_{0.15}Bi_{0.85}FeO_3$ film is epitaxially strained to the DSO substrate. **c,** Representation X-ray reflectivity scan for the determination of the layer thickness. The subscripts "p.c." and "o" refer to the pseudocubic and orthorhombic lattices of $BiFeO_3$ and $DyScO_3$, respectively.

2. STEM analysis of La^{3+}-rich and La^{3+}-poor layers

We investigated the composition of the *A*-site planes (*A* = Bi^{3+}, La^{3+}) in the region treated with a compressive force by electron energy-loss spectroscopy (EELS) and energy dispersive X-ray (EDX) spectroscopy in STEM mode. The STEM-EELS and STEM-EDX analyses were carried out using a probe-corrected FEI Titan Themis microscope equipped with ChemiSTEM technology and a CEOS Energy-Filtering and Imaging Device (CEFID) in combination with a direct electron detector (ELA, Dectris). The microscope was operated at an accelerating voltage of 300 kV.

The EELS data was obtained by setting a convergence and collection semiangles to 26 and 35 mrad, respectively. An energy dispersion of 0.75 eV per channel was chosen to record spectrum images of the La-L3,2 edge using 3 systematic samplings with a pixel dwell time of 1.0 ms, resulting in a total acquisition time of 12 s.

The La and Bi content of the $La_{0.15}Bi_{0.85}FeO3$ film was investigated by EDX spectroscopy by using the Bi−L and La-L edge intensities. The obtained atomic fractions are given in Table S1.

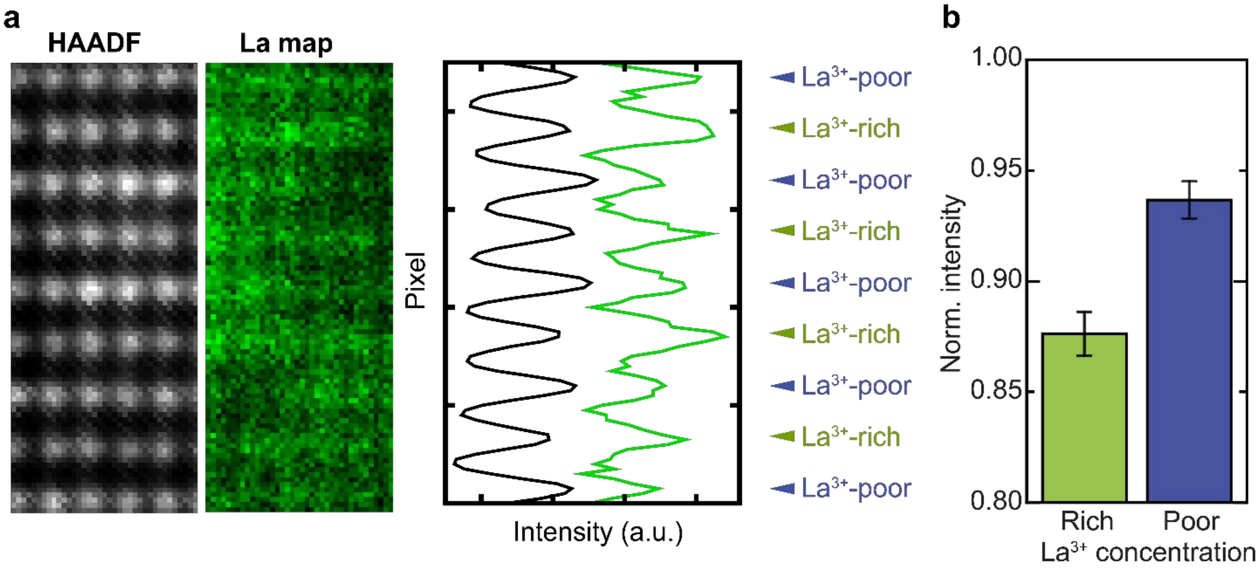

Fig. S2: STEM analysis of La^{3+}-rich and La^{3+}-poor layers. a, The HAADF-STEM survey image and corresponding EELS map of the La $L_{3,2}$ edge of the region treated by compressive force reveal the presence of alternating La^{3+}-rich and La^{3+}-poor layers. The averaged line profiles extracted across the vertical of the HAADF image and the La map are shown in black and green, respectively, and confirm a periodic intensity modulation in every other *A*-site layer. **b,** Quantitative estimation of the experimental La^{3+}/Bi^{3+} atomic-column intensities for the La^{3+}-rich and La^{3+}-poor layers in Fig. 2**b** was performed using a center-of-mass peak-finding algorithm and an iterative refinement of the fitted peaks by solving a least-squares minimization problem, as described in the methods section. The fitted intensities were normalized to the maximum atomic column intensity. This statistical analysis of the integrated peak values subtracts the effects of the cation shifts to the line profile in Fig. 2**b**, validating the existence of La^{3+}-rich and La^{3+}-poor layers. The error bars denote the standard error.

Table. S1: Energy dispersive X-ray analysis of the La/Bi content in the deposited $La_{0.15}Bi_{0.85}FeO_3$ film

Z	Element	Edge	Atomic fraction (%)	Atomic error (%)
57	La	L	16.43	1.72
83	Bi	L	83.57	1.72

3. Symmetry analysis of pristine, stressed and stressed-poled regions

We investigated the crystal symmetry of the pristine, stressed, and stressed-poled regions by performing two-dimensional fast Fourier transformations (FFTs) of the HAADF-STEM micrographs displayed in Figs. 1**a**, 2**a** and 4**a**, see Figs. S3**a**, S3**c**, and S3**e**, respectively. We find the *R3c* symmetry of the ferroelectric phase in the pristine and the poled regions, and the *Pnma* symmetry of the antipolar phase after force application. Simulated electron diffraction patterns of the respective phases are depicted in Figs. S3**b**, S3**d**, and S3**f**. Further, from our quantitative analysis of the lattice parameters by means of a peak-pair analysis at the interface between the pristine and the stressed regions, we obtain an increase in lateral and a decrease in vertical lattice parameters by 1% each when transitioning from the ferroelectric to the antipolar phase, see Figs. S4**c**, and S4**d**.

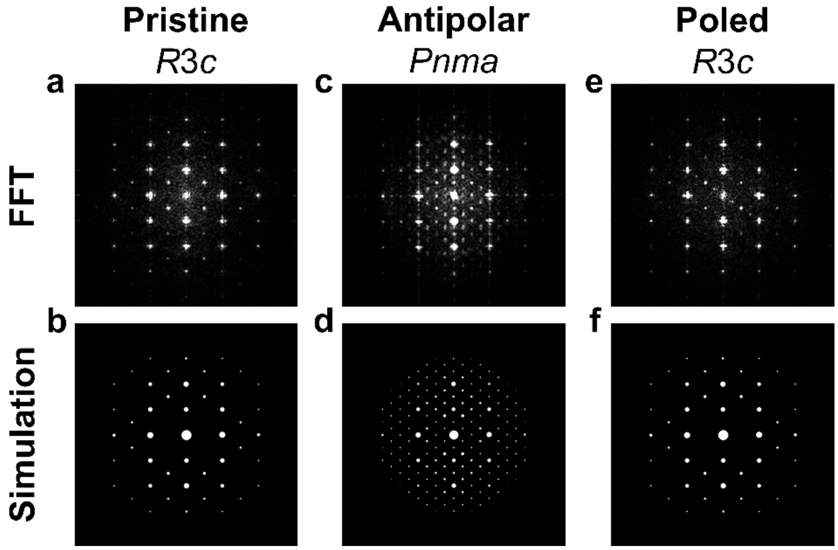

Fig. S3: FFT patterns extracted from the HAADF-STEM micrographs captured along $[100]_{p.c.}$ of **a**, a pristine region, **c**, a region treated with compressive force, and **e**, a region that had been first treated with compressive force and subsequently electrically poled. Electron-diffraction simulations of the R3c phase are depicted in **b** and **f**, respectively. The simulation of the antipolar *Pnma* phase is depicted in **d**.

4. Interface between *R3c* and *Pnma* phases

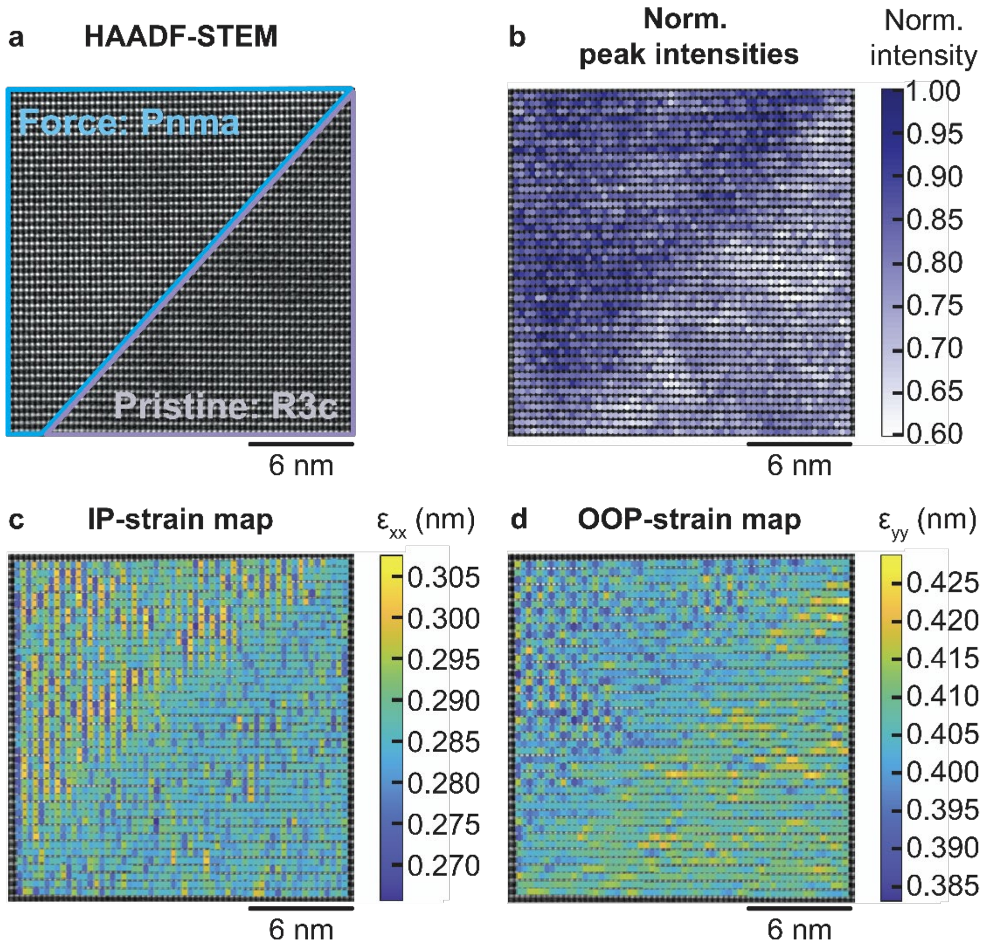

Fig. S4: Interface between the pristine region and a region that had been exposed to compressive force. **a**, HAADF-STEM micrograph of the interface inclined by about 45°. **b**, Fit of the normalized intensities of the Bi^{3+}/La^{3+} atomic columns plotted at their fitted coordinates superimposed on the HAADF image. Note that the stressed antipolar phase (left side) shows enrichment and depletion of La^{3+} distribution in alternating layers. Maps of the in-plane (**c**) and out-of-plane (**d**) interatomic distances for the Bi^{3+}/La^{3+} sublattices as obtained by peak-pair analysis. The stressed phase exhibits smaller out-of-plane and larger in-plane lattice parameters compared to the pristine phase.

5. Independence of topographic corrugation on the scanning direction

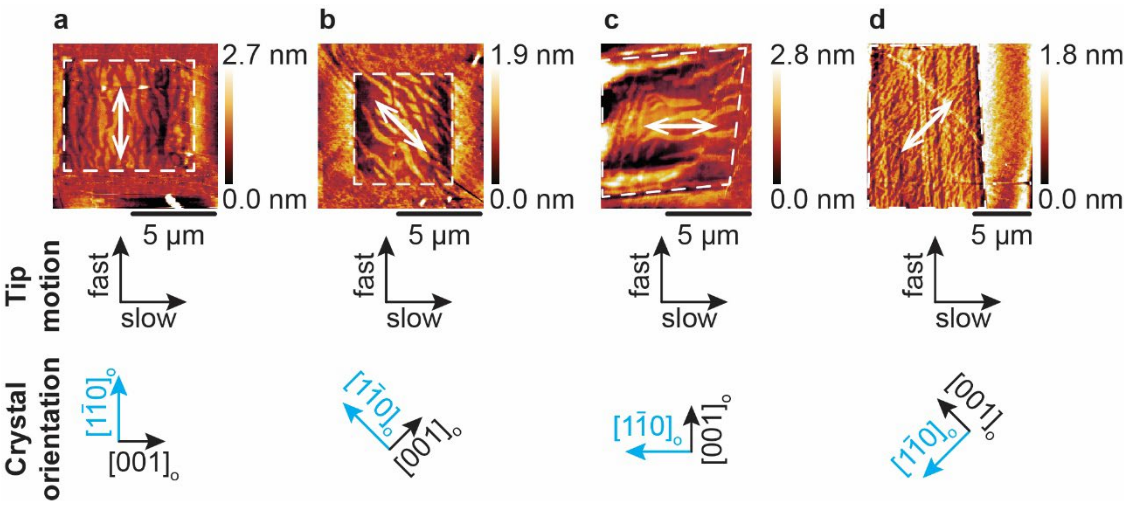

Fig. S5: Rotational dependence of the fast- and slow-scanning direction during compressive-force application. The sample is rotated by 45° for each image from **a**–**d**. The white double arrow marks the direction of the hill-and-valley channels. The coordinate systems below each subfigure show the fast- and slow-scanning direction and the crystal orientation. The $[1\bar{1}0]_o$-direction is highlighted in blue, as the corrugation channels always align with this crystallographic direction.

6. Absence of surface reconstruction

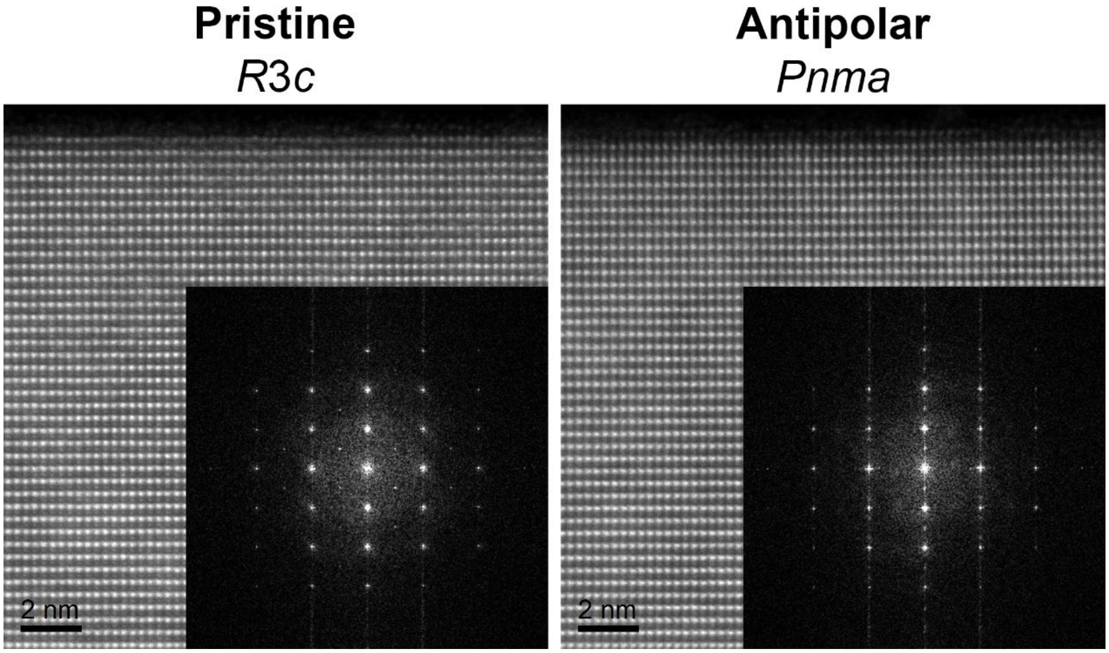

Fig. S6: Absence of surface reconstruction. HAADF-STEM micrographs of the film surface acquired along $[010]_{p.c.}$ of a pristine region (left side) and a region treated with a compressive force (right side). The corresponding FFT patterns are shown as insets and confirm the *R*3*c* phase and the antipolar *Pnma* phase for the pristine and compressed regions, respectively.

7. Histograms of LPFM maps

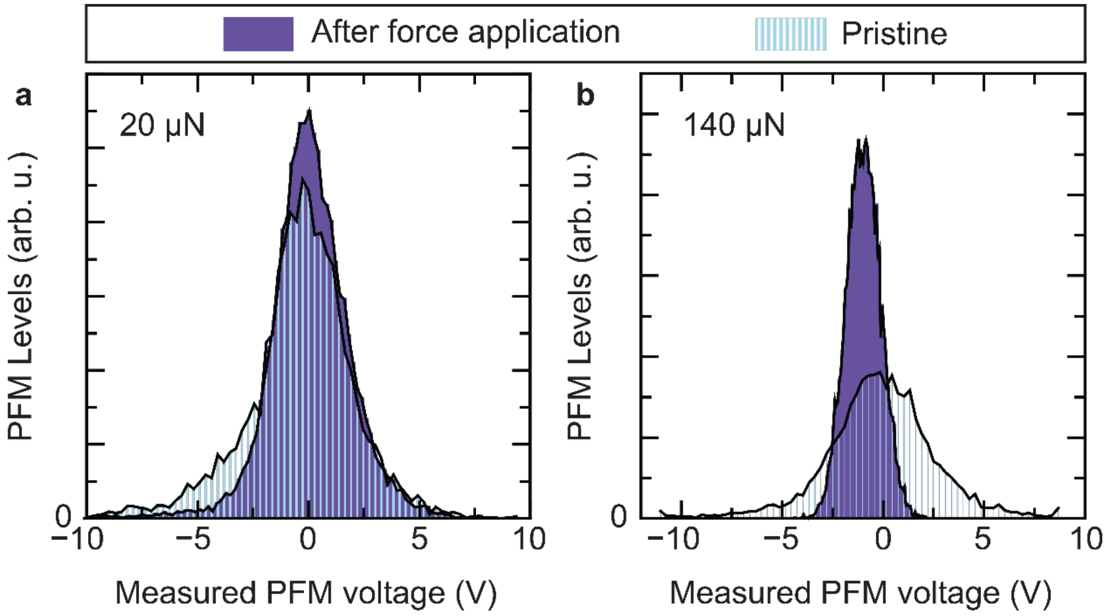

Fig. S7: Histograms of the LPFM images of (**a**) Fig. 3**a** and (**b**) Fig. 3**d**. The data colored in violet and blue corresponds to the pristine region and the region exposed to force, respectively.

8. Repeatable polar-antipolar phase transitions

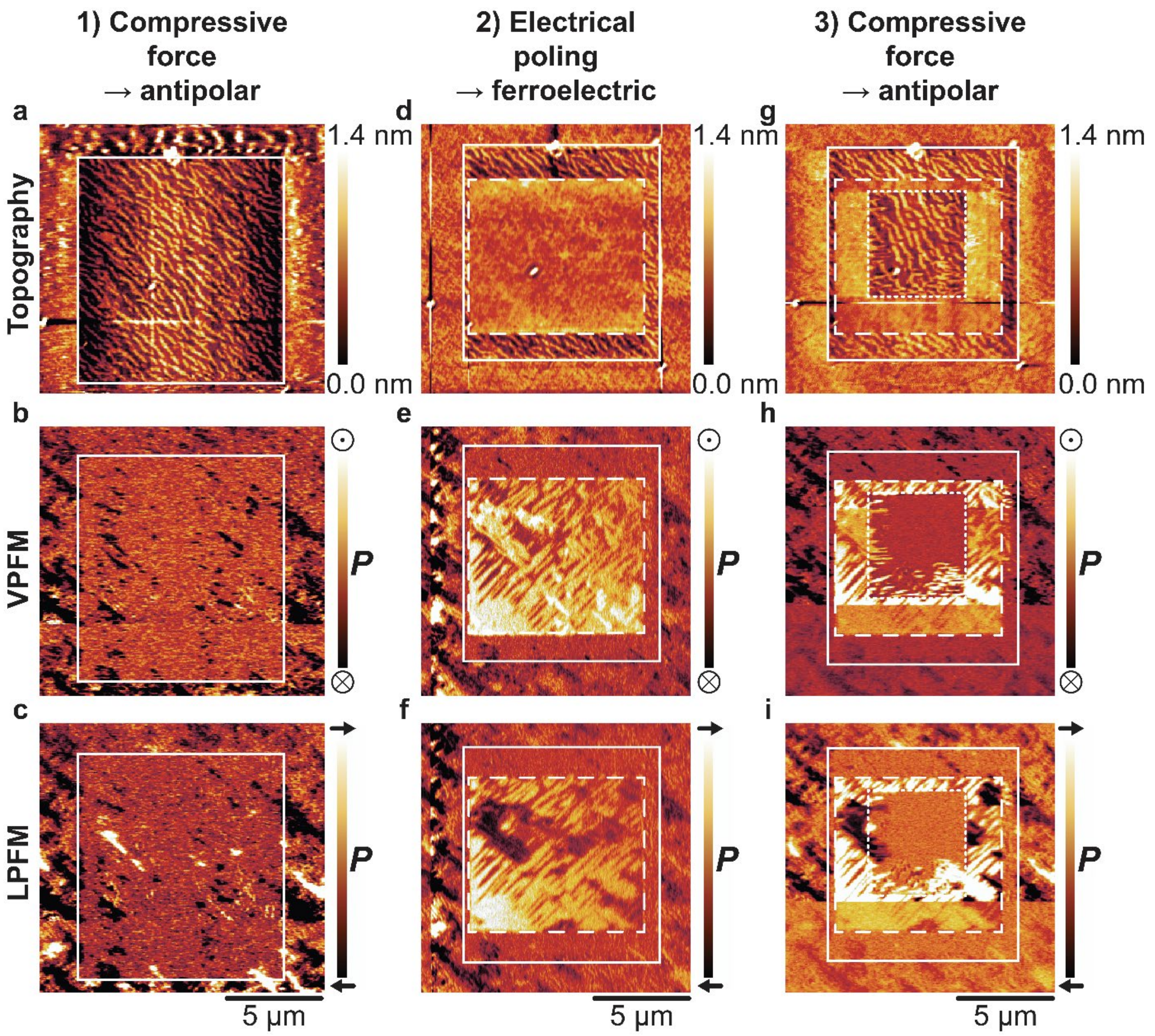

Fig. S8: Demonstration of the repeatability of the polar-antipolar phase interconversion. a–**c** show the topography, VPFM, and LPFM micrographs, respectively, after the first application of compressive force. **d**–**f** show the topography, VPFM, and LPFM micrographs, respectively, after consecutive electric-field application. **g**–**i** show the topography, VPFM, and LPFM micrographs, respectively, after a second round of compressive-force application. The solid squares indicate the region exposed during the first round of compressive-force application. The dashed squares mark the poled region. The dotted squares show where the compressive force was applied for the second time.

9. La^{3+}-substitution-dependent phase transition

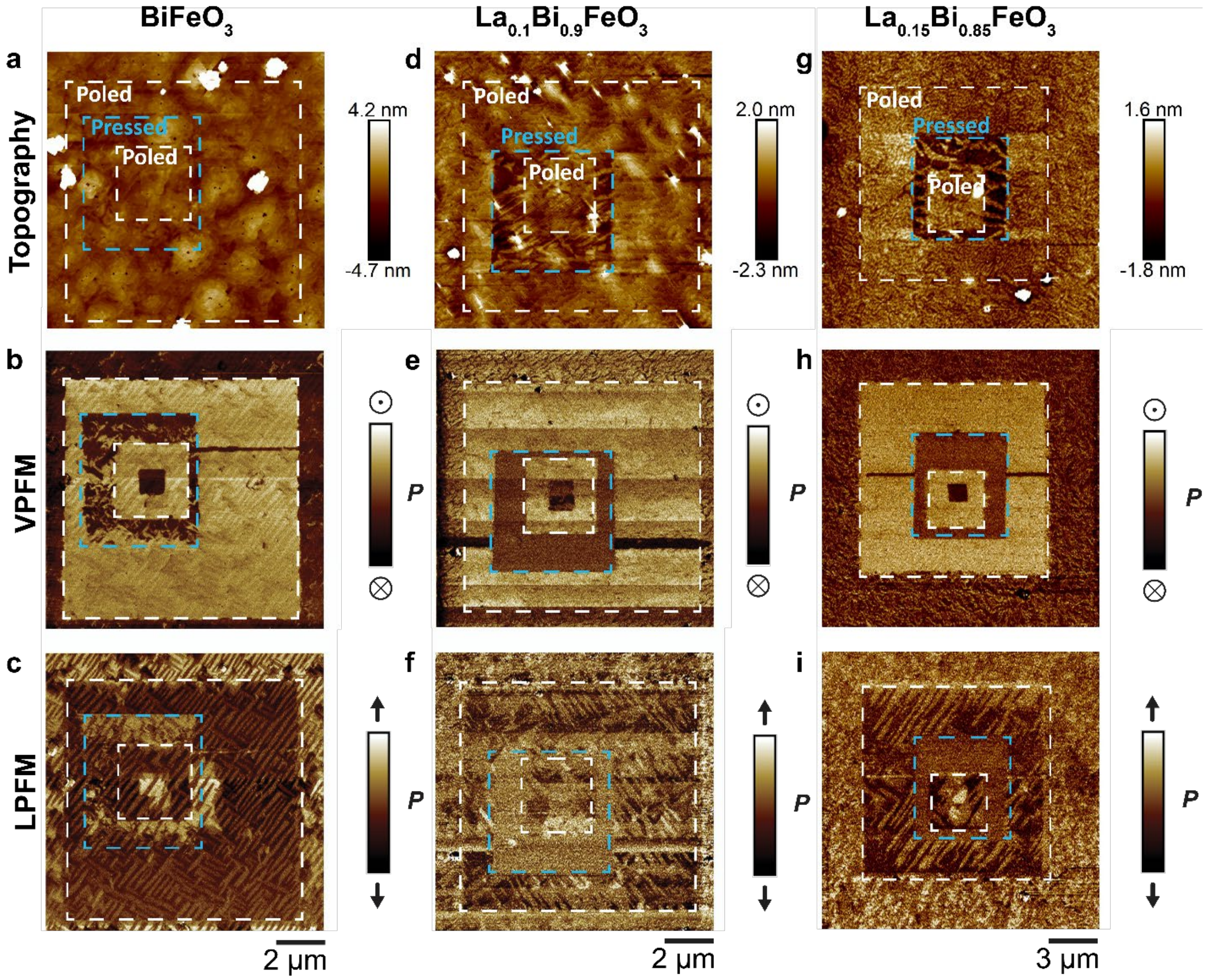

Fig. S9: La^{3+}-substitution-dependent phase transition. The topography, VPFM, and LPFM micrographs of **a**–**c** pure $BiFeO_3$ film without La substitution, **d**–**f** film with 10% La^{3+} substitution, and **g**–**i** film with 15% La^{3+} substitution. The outermost white-dashed squares indicate the regions with the first application of −10 V scanning probe-tip bias, which poles the out-of-plane polarization component upwards. The middle blue-dashed squares show the regions where the local pressure was applied. Only $La_{0.1}Bi_{0.9}FeO_3$ and $La_{0.15}Bi_{0.85}FeO_3$ show the characteristic topography change and elimination of PFM responses of the antipolar phase. The inner white-dashed squares show where the −/+ 10 V scanning-probe tip bias is applied after compressive-force application, where the ferroelectric phase is recovered.

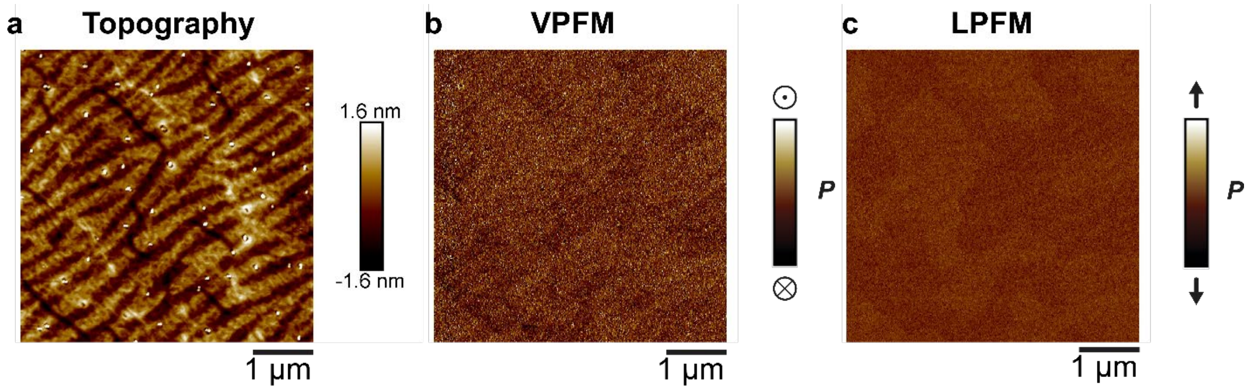

Fig. S10: a-c, The topography, VPFM, and LPFM micrographs of the pristine antipolar state of a $La_{0.2}Bi_{0.8}FeO_3$ film. No pressure nor electric field induced change in the piezoresponse is visible.

10. Stability of phase transition over time

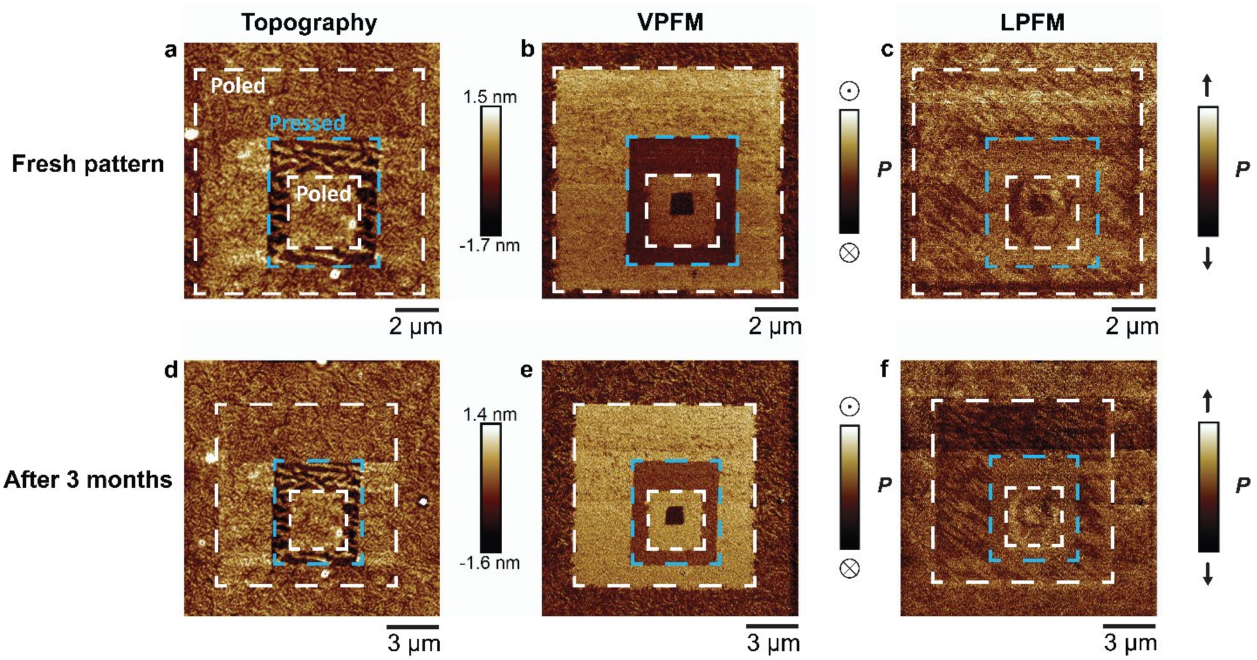

Fig. S11: The topography, VPFM, and LPFM micrographs of the same test pattern on a 100-nm-thick $La_{0.15}Bi_{0.85}FeO_3$ film when, **a-c,** it was freshly treated and**, d-f,** after three months. We did not observe any sign of degradation of the pressure-induced nor of the poled features over time.

11. Local interconversion of the polar and antipolar phases using conventional single-crystalline $SrTiO_3$ substrate

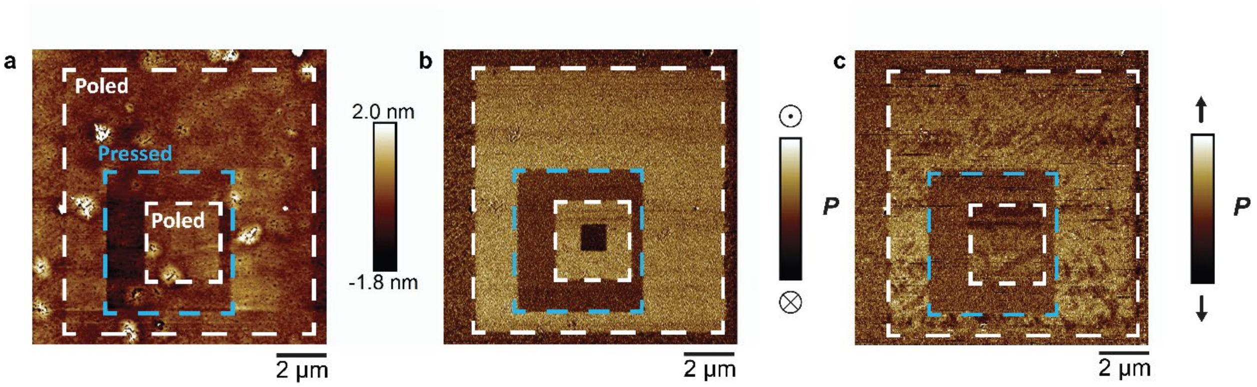

Fig. S12: Reversible control of the phase transition using $SrTiO_3$ substrate. a–c show the topography, VPFM, and LPFM micrographs, respectively, of a $La_{0.15}Bi_{0.85}FeO_3$ film grown on (001)-oriented $SrTiO_3$ substrate with $SrRuO_3$ buffer layer. The outermost white-dashed squares indicate the regions with the first application of −10 V scanning probe tip bias, which poles the out-of-plane polarization component upwards. The middle blue-dashed squares show the regions after local force treatment. The inner white-dashed squares show where the −/+ 10 V scanning probe tip bias is applied after the compressive force application.

12. Phase interconversion in sub-100 nm films

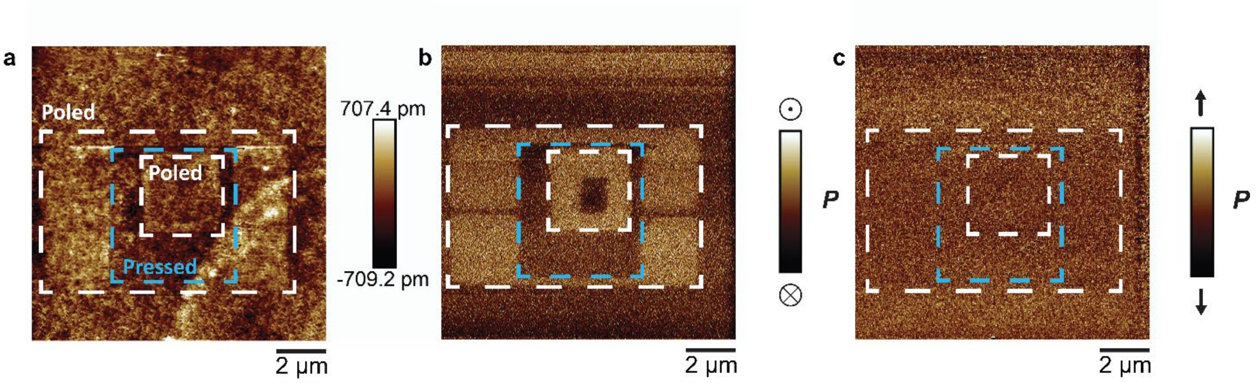

Fig. S13: Phase interconversion in sub-100 nm films. a–c show the topography, VPFM, and LPFM micrographs, respectively, of 20-nm-thick $La_{0.15}Bi_{0.85}FeO_3$ film grown on $(110)_o$-oriented $DyScO_3$ substrate with 8-nm-thick $SrRuO_3$ buffer layer. The outermost white-dashed squares indicate the regions with the first application of −10 V scanning-probe tip bias, which poles the out-of-plane polarization component upwards. The middle blue-dashed squares show the regions after consecutive local force treatment. The inner white-dashed squares show where the −/+ 10 V scanning probe tip bia was applied after the compressive force application. While the phase transition can be tracked by VPFM, the reduction of domain size prevents the observation of a change in contrast in the LPFM response.

13. Force calibration

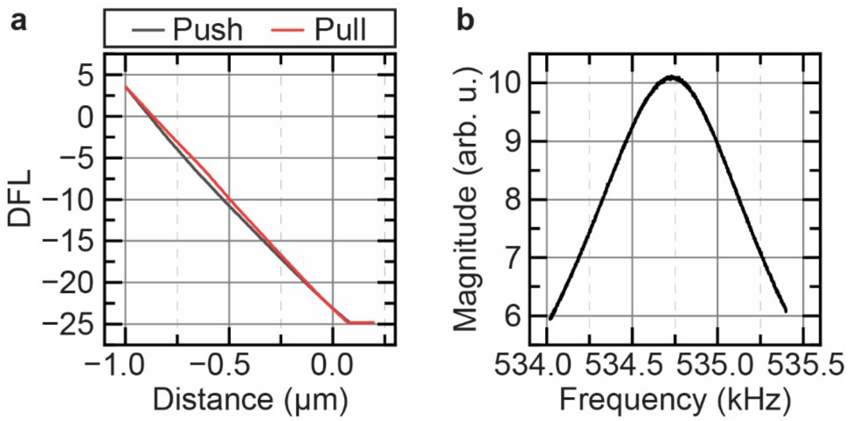

Fig. S14: a, Force-distance curve and **b**, resonance frequency of the diamond-coated tip. Push and pull denotes the action of the tip on the surface. DFL denotes the deflection of the tip measured by the four-quadrant photodetector.

In order to estimate the applied force, we need to derive the spring constant *k* of our tip. According to the manufacturer specifications, the resonance frequency of these tips should vary between 260 kHz and 630 kHz, and the force constant should range between 28 N/m and 91 N/m. We thus measure the resonance frequency ω of our tip, see Fig. S14**b**. We obtain a resonance frequency of 534.7 kHz. Since $\omega \propto \sqrt[4/3]{k}$, we can now estimate the force constant of our tip to 75 N/m. Knowing the force constant, we now calibrate the force applied to our deflection (DFL) measurement with the force-distance curve in Fig. S14**a**.
